# Prevalence of, Subtypes of, and the Role of Age in Incidental Appendiceal Neoplasms in Acute Appendicitis: A Single-Institute Study from Bahrain

**DOI:** 10.7759/cureus.60150

**Published:** 2024-05-12

**Authors:** Ahmed Saeed, Yomna Abuzaid, Maryam Hammad

**Affiliations:** 1 Surgery, Salmaniya Medical Complex, Manama, BHR; 2 Gynecology, Salmaniya Medical Complex, Manama, BHR; 3 Pathology, Salmaniya Medical Complex, Manama, BHR

**Keywords:** adenocarcinoma, carcinoid, neuroendocrine tumor, appendicectomy, acute appendicitis

## Abstract

Introduction: Primary appendiceal neoplasms (ANs) are rare entities that can present with acute appendicitis symptoms. Accurate diagnosis of these diverse subtypes is crucial for prognosis and proper management.

Aims and Objectives: This descriptive retrospective study aims to determine the prevalence and pathological subtypes of incidental ANs in patients presenting with acute appendicitis symptoms at Salmaniya Medical Center (SMC) in Bahrain between the period of January 2020 and March 2024. Particular focus was placed on investigating whether advanced age is a significant risk factor for these neoplasms.

Materials and Methods: The study included 38,643 patients (aged 15 years and above) who underwent appendectomy for suspected acute appendicitis during the study period. Demographic data, clinical diagnoses, preoperative imaging findings, histopathological reports, and management details were analyzed. Medical records of patients were retrieved from ISEHA system. Statistical analysis was done using Microsoft Excel.

Results: The results showed that 12 patients (0.04% per year) had different subtypes of appendiceal tumors. Neuroendocrine tumors were the most common, identified in nine patients (75%), including nine cases of well-differentiated neuroendocrine carcinoma (NEC). Other histopathological subtypes included low-grade appendiceal mucinous neoplasm (LAMN), adenocarcinoma, and goblet cell adenocarcinoma, each found in one patient. Additionally, two patients had confirmed appendiceal mucocele. The mean age of patients with ANs was 30 years (range: 19-52 years), and 66.6% were younger than 38 years.

Conclusion: These findings highlight the importance of considering ANs in the differential diagnosis of acute appendicitis, especially in older patients. Further research is warranted to confirm the role of age as a risk factor and guide clinical decision-making.

## Introduction

Appendiceal neoplasms (ANs) are a rare group of tumors that are often found incidentally in patients who have undergone appendectomy for acute appendicitis. The incidence of primary ANs has been reported to be around 0.7 to 1.7 per million per year in the USA, with a slight increase in the last decade to 0.97 per 100,000 person-years [1-3]. Patients with ANs often present with symptoms of acute appendicitis, such as right lower quadrant abdominal pain, nausea, anorexia, and vomiting. The pain is usually caused by peritoneal irritation due to the diseased appendix. Inflammation can progress to abscess formation, phlegmon, perforation, and generalized peritonitis in complicated cases [4]. Acute appendicitis is considered the most common presentation of these tumors, and they are often found incidentally intraoperatively or postoperatively in the histopathological specimen [5].

These neoplasms can be broadly divided into epithelial and non-epithelial lesions, as defined by the World Health Organization (WHO) [6]. Epithelial lesions include serrated lesions and polyps, mucinous neoplasms, and adenocarcinomas (further subdivided into colonic-type, mucinous, and goblet-cell adenocarcinomas). Non-epithelial lesions include neuroendocrine tumors (NETs).

The aim of this study was to determine the prevalence of ANs in a single center with a large volume of appendectomy following suspected appendicitis.

## Materials and methods

This retrospective study was approved by the Governmental hospitals research ethics committee with research approval number 39-310324. The study screened a total of 38,643 patients aged 15 years and above who presented with acute appendicitis and underwent appendectomy at Salmaniya Medical Center (SMC), the largest referral governmental hospital in Bahrain, during the period from January 1, 2020, to March 1, 2024. The diagnosis of acute appendicitis was confirmed by histopathological examination. Patients who underwent appendectomy with normal histopathology reports or other non-appendicular pathology were excluded from the study (as shown in the flowchart 1). Demographic data, clinical diagnosis, preoperative radiological imaging (US, CT scan, and MRI) reports and histopathological reports were collected and analyzed using descriptive statistics. The primary objective of this study was to determine the prevalence of ANs in this single-center patient population. Medical records of patients were retrieved from ISEHA system. Statistical analysis was done using Microsoft Excel and Minitab statistical software 17.1 edition.

**Figure 1:**
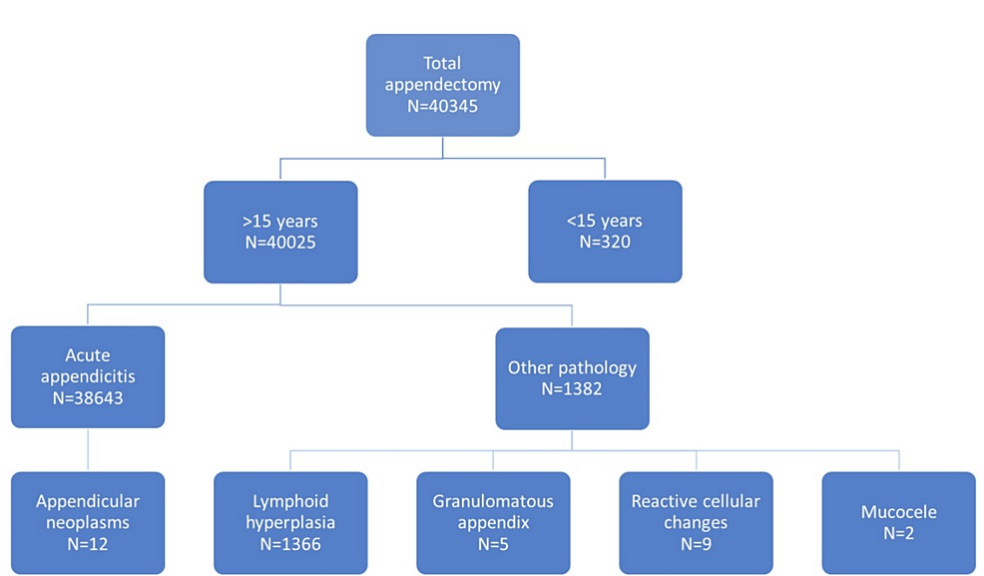
Distribution of our included and excluded study cohort

## Results

The study screened 38,643 appendectomy specimens for acute appendicitis, among which 12 cases of ANs were identified in our single center, representing a prevalence of 0.03% (3 per 10,000 appendectomies) per year. There were no other confirmed ANs in appendectomy specimens irrespective of the presence or absence of acute appendicitis in the final histopathology reports.

The demographic characteristics of these 12 patients are presented in Table 1. The male-to-female ratio was 1:1, with 6 patients in each group. The median age of patients with ANs was 30 years (± 9.4 SD). Histopathological features, T stage, and distribution of ANs are demonstrated in Table 2.

**Table 1:** Demographic data and clinical characteristics of the patients (N=12)

Age	Number (%)
15-39	10 (83.3%)
>40	2 (16.7%)
Gender	
Male	6 (50%)
Female	6 (50%)
Diagnosis modality	
Clinical	5 (41.6%)
US abdomen	2(16.7%)
CT abdomen	4 (33.3%)
Diagnostic laparoscopy	1 (8.4%)
Type of surgery	
Laparoscopic appendectomy	5 (41.6%)
Open appendectomy	7 (58.3%)
Further intervention	
Further surgery (right hemicolectomy)	2 (16.7%)
Without further surgery (right hemicolectomy)	10 (83.3%)
Expected diagnosis of neoplasm	
Clinically	0 (0%)
Imaging	2 (16.7%)
Intraoperative	1 (8.3%)
Non-expected	9 (75%)

**Table 2:** Histopathological features, T stage, and distribution of ANs (N=12) LAMN: Low-grade appendiceal mucinous neoplasm; HAMN: High-grade appendiceal mucinous neoplasm; AN: Appendiceal neoplasm

ANs subtypes	N (%)
Neuroendocrine tumor	9 (75%)
Well-differentiated (G1)	9 (100%)
Moderately differentiated (G2)	
Poorly differentiated (G3)	
Mitotic rate	
<2	9 (100%)
Ki-67%	
<2%	9 (100%)
Mucinous neoplasm of appendix	
LAMN	1 (8.3%)
HAMN	0
Adenocarcinoma	1 (8.3%)
Well-differentiated	0
Moderately-differentiated	1 (100%)
Poorly differentiated	0
Goblet cell adenocarcinoma	1 (8.3%)
Well-differentiated	0
Moderately-differentiated	1 (100%)
Poorly differentiated	0
Location	
Proximal half	2 (16.6%)
Distal half	6 (50%)
Tip of appendix	3 (25%)
Diffuse	1 (8.4%)
Size	
<2cm	9 (75%)
2-4cm	1 (8.4%)
>4cm	2 (16.6%)
Perforated	1 (8.4%)
Non-perforated	11(91.6%)
Lymphovascular invasion	2 (16.6%)
Perineural invasion	2 (16.6%)
Margin	
Involved	0
Not involved	12 (100%)
Regional lymph nodes	0
Immunohistochemistry	
Chromogranin	9 (75%)
Synaptophysin	6 (50%)
CDX2	1(8.3%)
T stage	
pTis	1 (8.3%)
pT1	3 (25%)
pT2	1 (8.4%)
pT3	6 (50%)
pT4a	1 (8.3%)

The diagnosis of acute appendicitis was made clinically in five cases (41.6%), while other diagnostic modalities, such as imaging, were used in (58.4%) seven cases. Preoperative imaging was able to suspect the diagnosis of AN in only (16.6%) of cases, and intraoperatively, the diagnosis was suspected in only one case (8.3%). In the vast majority of ANs, nine cases (75%) were not suspected either preoperatively or intraoperatively.

The majority of patients were treated with appendectomy alone in 10 cases (83.3%), while two cases of adenocarcinoma and goblet cell adenocarcinoma underwent additional right hemicolectomy as follow-up surgery (Table 1 and 3). Most cases (7; 58.4%) were managed with open appendectomy, while the remaining underwent laparoscopic appendectomy without conversion. All patients were discharged uneventfully, with no complications reported during the postoperative period or follow-up with a mean period of 20.8 months (± 17.6 SD).

**Table 3:** Clinicopathological features of ANs patients and management (N=12) AN: Appendiceal neoplasm

No	Age	Sex	Diagnosis	Operative procedure	Operative findings	Histopathology	Size, T-stage	Location	Further treatment
1	30	F	Appendicitis	Lap App	AA	Net G1	0.8 cm T3	Proximal half	No
2	34	M	Appendicitis	Lap App	AA	Net G1	1.2cm T3	Tip	No
3	22	M	Appendicitis	Open	AA	Net G1	0.9 cm T3	Distal half	No
4	28	F	Appendicitis	Lap App	AA	Net G1	1 cm T3	Tip	No
5	52	M	Appendicitis	Open	AA early mass forming	Net G1	0.8 cm T1	Distal half	No
6	19	F	Appendicitis	Lap App	AA	Net G1	0.6 cm T3	Distal half	No
7	30	F	Appendicitis	Lap App	AA	Net G1	0.7 cm T1	Distal half	No
8	24	F	Appendicitis	Lap App	AA	Net G1	0.7 cm T3	Proximal half	No
9	30	M	Appendicitis	Lap App	AA	Net G1	1 cm T2	Tip	No
10	38	F	Appendicitis	Open	Huge mucocele	LAMN	8cm Tis	Diffuse	No
11	38	M	Appendicitis	Open	Perforated mass forming appendix	Adenocarcinoma	2.5cm T4a	Distal half	Right hemicolectomy
12	44	M	Appendicitis	Open	AA, gangrenous tip abscess	Goblet cell adenocarcinoma	4cm T3	Distal half	Right hemicolectomy

Neuroendocrine tumors (NETs) were the most common ANs, found in nine patients (75%), with a female predominance (5; 55.5%) (Tables 1 and 3). The age range of NET presentation was 19 to 52 years, with the majority (8 out of 9; 88.8%) occurring in the second and third decades of life. The mean age of appendiceal neuroendocrine tumor (A-NET) patients was 29.9 years (± 9.5 SD). All NETs were well-differentiated (G1) according to the American Joint Committee of Cancer (AJCC) classification. The majority (8; 88.8%) of NETs were less than 1 cm in size, and seven patients (77.7%) were located in the tip and distal half of the appendix (Table 3). All NETs, regardless of T stage, achieved clear tumor-free margins, including six cases (66.6%) with T3 invasion into the subserosa.

The remaining ANs included one case of low-grade appendiceal mucinous neoplasm (LAMN), one case of moderately differentiated adenocarcinoma, and 1 case of moderately differentiated goblet cell adenocarcinoma (Table 2). The LAMN case was suspected intraoperatively as a mucocele, and the adenocarcinoma cases were suspected preoperatively on CT imaging. Both adenocarcinoma cases were male, while the LAMN case was female.

**Figure 2:**
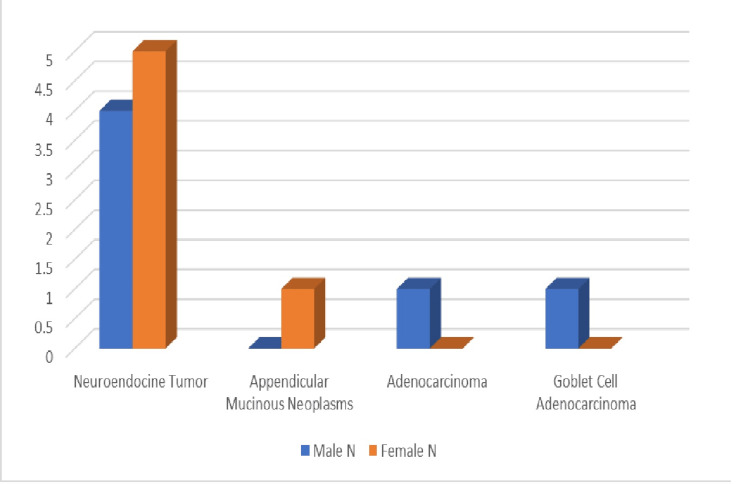
Distribution of ANs by sex AN: Appendiceal neoplasm

The distribution of ANs by sex is shown in Figure 2. The epithelial ANs, such as adenocarcinoma and goblet cell adenocarcinoma, were more common in the middle-aged group above 35 years.

## Discussion

Acute appendicitis is the most common surgical diagnosis of acute abdomen in children and adults. The incidence of appendicitis peaks in the second and third decades of life [7]. Appendectomy is the most common emergency general surgical procedure worldwide and is estimated around 50000 to be performed in the UK yearly [8]. In our center SMC, appendectomy is the most commonly performed emergency abdominal surgical procedure with an estimate of about 10,000 a year. Interestingly, this is a relatively high number of appendectomies performed in a country of our size, in comparison to a large country like the UK. It could be referred to our population demographic figure with a large number of youngsters [9]. Captivatingly, the prevalence of ANs in our single center population (0.03%) was far less than the international figures which represent only a short study period in a single center excluding other referrals, and community hospitals that may encounter similar histopathological findings of ANs. Furthermore, the genetic characteristic of our race with a predominantly young population could be contributing to the rare predisposition of such scarce neoplasms.

Appendicular tumors are still one of the rarest neoplasms that are found incidentally after appendectomy in histopathological specimens [6]. The age presentation of ANs varies depending on the subtypes of neoplasms as reported in the literature. This is attributed to the predominance of A-NETs in our cohort, which presented at a young age with acute appendicitis. The most common age of presentation of NET was in the second and third decades concordant with other reports [10]. Nonepithelial ANs are most pronounced after the age of 40 which is almost consistent with our results [11]. Although appendiceal adenocarcinoma is more frequently found amongst men in the sixth to seventh decades of their lives, recently, they have been diagnosed at an earlier age [12]. This was proven with our observation of male predominance in our small patient group. However, our finding negates the assumption of common adenocarcinoma development in the old age group. This could be attributed to the presentation of such patients with acute appendicitis symptoms in their middle age, fourth decade rather than sixth or seventh decades. Therefore, it is crucial to study the postoperative specimens to detect such neoplasms and categorize their diverse histology to determine their prognosis and proper management [13,14]. The prevalence of ANs in our single center was very low about 0.03% per year in comparison to the international figures between 0.2% and 2.5% [6].

ANs had diverse subtypes and pathological classifications, which underwent several revisions reaching the current consensus in practice of the 2019 WHO classification of tumors of the digestive system. This classification categorizes particularly the mucinous ANs and pseudomyxoma peritonei (PMP) according to the Peritoneal Surface Oncology Group International (PSOGI) Executive Committee [2,10,15].

The 2019 WHO classification broadly divides appendiceal tumors into epithelial and non-epithelial neuroendocrine neoplasms. The epithelial tumors of the appendix split into the following categories: hyperplastic polyp, sessile serrated lesion with or without dysplasia, LAMN, HAMN, adenocarcinoma, undifferentiated carcinoma, goblet cell adenocarcinoma, and NETs [2,4,16,17].

The most commonly occurring appendiceal tumor subtype is neuroendocrine appendiceal tumor [10]; this is in line with our results. The incidence rate in our cohort was 75% of all other subtypes, while it ranges from 35% to 85% of all appendiceal tumors in the largest national cancer institute patient series available through the SEER database [10,11]. The patients are discovered to be in the 20s and 30s with mostly acute appendicitis symptoms as in our cohort. It occurs more commonly in females evidenced in our study, with five out of nine patients and as reported by some authors. Appendicular NETs are considered the first or second rank of all gastrointestinal NET GI-NET followed by small bowel and rectum [18]. A-NETs present at a younger age than other GI-NETs [2].

The majority of cases presented with acute appendicitis picture or nonspecific symptoms of abdominal pain and rarely iron deficiency anemia or weight loss unless advanced poor differentiated tumor. This is concordant with our results as all presented with acute appendicitis symptoms. They rarely presented with abdominal distention which represents advanced peritoneal dissemination [10,11].

A-NETs secrete serotonin and other bioactive materials. However, they seldom present with carcinoid syndrome unless they metastasize to the liver in advanced cases only. Therefore, they are usually found incidentally intraoperative or postoperatively [19]. They are classified according to WHO 2019 into well-differentiated NETs, poorly differentiated neuroendocrine carcinomas (NECs), and mixed neuroendocrine-non-neuroendocrine neoplasms (MiNENs) [4].

Appendicular tumors are commonly located at the tip of the appendix with less than 1 cm tumor size [20]. This was almost the case in our cohort, with seven cases of tumors found in the tip and distal half of the appendix (77.7%). Eight out of nine were 1 cm and less with only one case with 1.2 cm tumor size. This result is comparable with other reports. The management of A-NETs <2 cm in size is appendectomy alone, which is considered curative with a negative margin (R0); therefore, no further follow-up up was recommended. This approach of management was adopted at our center. However, for tumors > 2cm or with high-risk features (poor differentiation, involving the base or invading mesoappendix or lymphovascular invasion), right hemicolectomy was recommended [14]. A-NETs carry the best survival rate of all appendicular tumors. The 10-year survival rate of A-NETs ranges from 90-100% based on tumor size. They also had a low risk of recurrence near 0% in <2 cm tumors [21]. Thus, appendectomy alone is considered sufficient management. The follow-up for those tumors is usually by monitoring Chromogranin-A and serotonin levels as an indicator of treatment response or recurrence [22].

Appendicular serrated lesions did not invade beyond lamina propria or distort mucosal architecture. Therefore, appendectomy with a negative margin is considered enough treatment [4,23].

Appendicular mucinous neoplasms AMNs compose the mainstream of epithelial lesions. They arise from neoplastic goblet cells [24]. They commonly present with peritoneal carcinomatosis (jelly belly) from a ruptured mucin-filled appendicular tumor called PMP. The clinical pictures of PMP are quite challenging as are usually presented with incidental findings during appendectomy or laparoscopy and laparotomy for other reasons. Abdominal pain and distention with rapid weight loss signify the advancement of the disease. Yet they also presented as unruptured tumors [25]. 

LAMN is characterized by loss of the muscularis mucosae or lamina propria, a pushing pattern of growth or diverticulum formation, submucosal fibrosis, dissection of acellular mucin into the appendiceal wall, or mucin/neoplastic mucinous epithelium outside of the appendiceal wall. In HAMN, high-grade cytologic atypia is encountered in the absence of infiltrative invasion of the appendix and will not be associated with metastatic disease. In the case of unruptured LAMN or HAMN, treatment with appendectomy including mesoappendix is sufficient [25]. Due to the indolent nature of the disease, it tends to have very low recurrence rates.

In terms of ruptured mucinous disease, PSOGI classifies them as acellular mucin, low-grade mucinous carcinoma peritonei, high-grade mucinous carcinoma peritonei, and high-grade mucinous carcinoma peritonei with signet-ring cells [4]. Cytoreductive surgery and heated intraperitoneal chemotherapy (CRS/HIPEC) is the standard surgical approach with significant survival benefits [26].

Mucinous adenocarcinoma of the appendix (MACA) and colonic-type appendiceal adenocarcinoma are subdivided by a grading system into well, moderate, and poorly differentiated. The moderate and poorly differentiated lesions usually metastasize through lymphatic with nodal involvement. The presence of signet ring cells in adenocarcinoma carries the poorest prognosis. A formal right colectomy in localized appendiceal adenocarcinoma is recommended to adequately resect and stage the disease. In moderate and poorly differentiated adenocarcinoma, adjuvant systemic chemotherapy follows surgery. In females, bilateral salpingo-oophorectomy is recommended regardless of age due to similar histological potential for mucinous dissemination and advanced metastasis yet no consensus on such practice [27].

Appendicular goblet-cell adenocarcinomas are extremely rare tumors that contain both neuroendocrine and mucinous cells. They similarly graded histologically into well, moderate, and poorly differentiated lesions. They are treated the same as mucinous adenocarcinoma with formal right colectomy [28]. In our study, moderately differentiated goblet cell adenocarcinoma was found and treated with right hemicolectomy due to a large size >2 cm and lymphovascular and perineural invasion. 

Tumor markers, including CEA, CA19-9, CA-125, chromogranin-A, and/or 5-HIAA, may help reach the diagnosis of ANs if significantly elevated [29,30]. Though they are requested more frequently after postoperative diagnosis. It also warranted undergoing a complete colonoscopy in such a sitting, especially since synchronous colonic pathology is at a higher rate in such a group [31].

The main limitations of the study include a small sample size and short follow-up duration. This study represented the incidence of AN in the main governmental and referral hospital in Bahrain with the limitation of the unavailability of advanced CRS and HIPEC for the management of mucinous ANs.

## Conclusions

Appendiceal cancers are among the rarest tumors encountered incidentally during postoperative histopathological examination. Consistent with the existing literature, neuroendocrine neoplasms emerged as the most common appendicular tumors across the case series analyzed.

An intriguing observation in our study was the increased proportion of advanced adenocarcinoma and goblet cell adenocarcinoma of the appendix, specifically within the middle-aged patient cohort. This finding underscores the importance of maintaining a high index of suspicion for appendiceal malignancies, particularly in middle-aged individuals.

Comprehensive preoperative imaging and meticulous intraoperative inspection of the appendix are crucial to guide the surgical approach and maximize the chances of achieving a curative R0 resection. Early recognition of suspicious appendicular lesions, followed by appropriate surgical management, is key to optimizing oncological outcomes in these rare but clinically relevant tumors.
